# Comparison of psychosocial factors between patients with benign fasciculations and those with amyotrophic lateral sclerosis

**DOI:** 10.4103/0972-2327.53079

**Published:** 2009

**Authors:** Sandeep S. Rana, Carol J. Schramke, Amandeep Sangha, Aryn C. Karpinski

**Affiliations:** 1 Department of Neurology, Allegheny General Hospital, Pittsburgh; Philadelphia, PA, The Ohio State University, Columbus, OH, USA; 2 Department of Neurology, Drexel University College of Medicine, Philadelphia, PA, The Ohio State University, Columbus, OH, USA; 3 The Ohio State University, Columbus, OH, USA

**Keywords:** Amyotrophic lateral sclerosis, muscle disorders, neuropsychology

## Abstract

In this retrospective study, we compared the initial presentation of patients who were eventually diagnosed with either benign fasciculations (BF) or amyotrophic lateral sclerosis (ALS). We found a significantly higher number of patients with BF reporting a past history of psychiatric symptoms, life stressors, and concurrent psychosomatic symptoms. There was no difference between the two groups in patient report of current anxiety or depression symptoms. These findings support our hypothesis that BF are a manifestation of psychological distress due to somatization and that reviewing psychosocial history is important when patients are being evaluated for fasciculations. Patients seeking medical attention for fasciculations and who do not report a history of underlying psychiatric or psychosomatic disorders should be followed closely as fasciculations have been reported to be a presenting feature of ALS.

## Introduction

Neurologists often encounter patients whose chief complaint is of frequent and persistent fasciculations, which may or may not be associated with cramping. Some of these patients have associated weakness and atrophy of muscles due to amyotrophic lateral sclerosis (ALS), or some other chronic peripheral neuropathic disorder (e.g., radiculopathies). In others, despite an extensive work-up, no definite etiology can be found for the fasciculations and the diagnosis of benign fasciculation (BF) syndrome is given.

Fasciculations can be the presenting feature of ALS in some patient's[1–4] and patients with BF may become anxious when they read these reports on the Internet or other sources. In our patient population who were eventually diagnosed with BF, we noted considerable somatic preoccupation and somatic complaints during the initial examination. To the best of our knowledge, there have been no published studies documenting the incidence of psychosocial factors in patients with BF and no studies examining whether or not these patients present differently from patients with ALS.

The objective of this study was to retrospectively review patients' reports of their psychiatric history, life stressors, and psychosomatic complaints at the time of the initial clinic visit and compare the prevalence of these variables in patients who were eventually diagnosed with either BF or ALS. We hypothesized that patients diagnosed with BF would present with complaints and clinical histories consistent with greater psychopathology as compared to patients with ALS.

## Materials and Methods

Patients diagnosed with BF through our clinic were identified by searching computerized billing records from 2003 through 2007 for this diagnostic code. This yielded a total of 13 patients who had presented with fasciculations and whose neurological examination, blood workup (including serum electrolytes and thyroid function tests), as well as nerve conduction studies/electromyography were normal. Only these patients were included in the BF group. We then reviewed our database, which contained all the patients seen through our ALS clinic and identified 15 patients who were seen during that same time period and were of similar age as those in the BF group. The diagnosis of ALS had been on the basis of the El Escorial criteria.[5]

All patients at their initial visit to our clinic are asked to fill out a detailed questionnaire to document co-morbidities and record a multisystem review. A consultation report of the initial visit is dictated by the evaluating neurologist and a record of this is also available. Our research assistant was asked to review these documents for evidence of: 1) psychosomatic symptoms, such as sleep disturbance, headaches/migraines, irritable bowel syndrome, nausea, heartburn/indigestion, loss of appetite, anorexia, weight loss or weight gain, and fatigue;[6] 2) life stress e.g., divorce, recent childbirth, etc.; 3) past history of psychiatric illness, including any report suggestive of past anxious or depressive symptoms; 4) current anxiety complaints, such as nervousness, panic attacks, etc. and 5) current depression complaints, such as sadness, low mood, moodiness, etc. All records were rated blindly; the research assistant was not told the purpose of the study and was not familiar with the expected associated symptoms of BF or ALS.

The data were analyzed with SPSS 14.0. We used Chi-square tests, Fisher's exact tests, and *t*-tests, as appropriate, to assess the differences between groups. We hypothesized that the BF group would have a higher incidence of all of these variables of interest. It has been suggested that age and gender can influence the incidence of somatic complaints, with women and older adults being more likely to have somatic complaints. To ensure that the BF and ALS groups were adequately matched with regard to age and gender, a *t*-test and chi-square analysis were conducted to test for between- group differences.

## Results

In the BF group, the youngest patient was 20 and the oldest was 59; the mean age was 41.7 ± 10.7 (SD) years. In the ALS group the ages ranged from 29 to 58 years and the mean age was 46.3 ± 8.7 years. An independent-sample *t*-test showed that this difference in the mean ages was not statistically significant (*P* = 0.25). Eleven out of 13 (85%) patients in the BF group and 10 out of 15 (67%) in the ALS group were male. Chi-square analysis showed that this difference in the proportion of males was not significant (*P* = 0.40).

The prevalence of psychosomatic symptoms, life stress, history of psychiatric illness, current anxiety complaint, and current depression complaints were then compared between the groups. These data are summarized in Table 1. Chi-square analyses found significantly higher prevalence of psychosomatic complaints (*P* = 0.02), life stress (*P* = 0.03), and past history of psychiatric illness (*P* = 0.02), in BF patients. There were no significant between-group differences in the reports of current anxiety (*P* = 0.41) and current depression (*P* = 0.19). These results are summarized in Table 2.

**Table 1:** Rating criteria

Psychosomatic symptoms	Sleep disturbance, headaches/migraines, irritable bowel syndrome, nausea, heartburn/indigestion, loss of appetite, anorexia, weight loss or weight gain, fatigue.
Life stress	Any report of life stress (e.g., divorce, worries about children, financial concerns, etc).
History of anxiety or depression	Any direct report suggestive of past history of symptoms or behavior related to anxiety or depression.
Current anxiety	Any direct report of current anxiety or anxiety-related symptom (e.g., nervousness, panic attacks, etc).
Current depression	Any direct report of current depression or depression-related symptom (e.g., sadness, low mood, moodiness, etc).

**Table 2:** Group differences between the ALS group and the BF group in the distribution of major variables

Variable name	^1^ALS (n = 15)		^2^BF (n = 13)		*XX*^2^	*P*
				
	Yes %	No %	Yes %	No %		
Psychosomatic symptoms	40	60	84.6	15.4	5.81	*0.016
Life stress	20	80	61.5	38.5	5.04	*0.025
History of anxiety or depression	26.7	73.3	69.2	30.8	5.07	*0.024
Current anxiety	26.7	73.3	38.5	61.5	0.68	0.411
Current depression	40	60	23.1	76.9	1.74	0.187

ALS = Amyotrophic lateral sclerosis; BF = Benign fasciculation.

* *P* < 0.05 indicates statistical significance

In the BF group, long-term follow-up of over 2 years was available in six patients. These patients had been treated with selective serotonin reuptake inhibiting agents and had received reassurance that they did not have ALS. All these patients were documented to have done well, with the fasciculations subsiding over time. None of the other seven patients in the BF group returned for follow-up visits despite having been asked to do so. Since it is very likely that if they had progressed to ALS they would have been referred back for treatment to our ALS clinic, we believe that there was no progression of their symptoms.

## Discussion

In our study, a significantly higher number of patients with BF had a history of psychiatric illness. Their fasciculations generally occurred at a time of significant life stress, such as divorce or some other traumatic event. These patients also reported psychosomatic symptoms such as irritable bowel syndrome, headaches, heartburn, anorexia, and weight loss or weight gain. Such patients appear to be preoccupied by the presence of fasciculations and seek multiple medical evaluations. Our findings suggest that BF may be a sign of difficulty coping with life stress and a form of somatization. Those of our patients with BF who were followed up did well with reassurance and serotonin reuptake inhibitors; none were documented to have developed ALS.

The number of patients reporting current anxiety and depression was comparable in the two groups. The percentage of ALS patients in our study reporting depression was relatively low, but this is consistent with other studies on ALS patients reported in the literature.[7,8] The fact that BF patients did not also have high percentages of current anxiety or depression may also be consistent with our understanding of patients with somatization disorders: These patients are more likely to both develop physical symptoms in response to psychological stress and to report them, and are less likely to recognize and report psychological distress. We hypothesize that patients with BF differ from those with ALS in that they have a personality type that makes them prone to psychosomatic illness and to focus on their body when they are stressed or exposed to a traumatic situation.

We found that in the group of patients with BF, there was a predominance of young males from the higher socioeconomic strata holding positions of responsibility. Although this aspect was not systematically examined in this study, eight patients (61.5%) in the BF group reported being physically active in sports (e.g., weight training, long-distance running, etc.) or were in a profession requiring a higher than average level of physical fitness (e.g., police force.) It is interesting to note that ALS has also been reported to have a higher incidence in athletic individuals,[9] although the pathophysiology of this link remains unclear.

The limitation of our study was its retrospective design, the relatively small sample size and the fact that a structured clinical interview was not used for assessing psychosocial stressors. We also acknowledge that although our data has shown an association between psychosomatic illnesses and BF, a cause-effect relationship cannot be deduced with certainty.

## Conclusion

One of the goals of this study was to provide guidance to physicians when they are confronted with patients having fasciculations and are apprehensive that it may be a sign of evolving ALS. The diagnosis of ALS is based on abnormalities detected on neurological examination and confirmation of motor denervation on electromyography, both of which are easy to demonstrate when the disease is advanced. In early disease, however, the diagnosis can be challenging. Our study suggests that, in addition to the lack of neurological deficits and the absence of abnormalities on electromyography, a history of psychiatric disorders and psychosomatic illnesses in persons experiencing increased burden of life stressors could be viewed as evidence supporting the diagnosis of BF. However, as a corollary, in the absence of these factors in a patient otherwise fitting the diagnosis of BF, a cautious approach would be advisable. These patients should be followed more closely and may need serial electromyography,[10] because there have been reports of cases where fasciculations were the presenting feature of ALS.
